# Experiencing obesity: trajectory and fruitless sacrifices to lose weight prior to bariatric surgery

**DOI:** 10.1590/1980-220X-REEUSP-2026-0209en

**Published:** 2026-09-25

**Authors:** Blessing Itzel Palacios-Meneses, Sarah Bak-Geller Corona, Araceli Jiménez-Mendoza, Carlos Enrique Yepes Delgado, Antonio González Chavez, Laura Olivia Pech Rueda, Sandra Hernández-Corral

**Affiliations:** 1 Universidad Nacional Autónoma de México, Facultad de Estudios Superiores Iztacala, Estado de México, México.; 2 Universidad Nacional Autónoma de México, Instituto de Investigaciones Antropológicas, Ciudad de México, México.; 3 Universidad Nacional Autónoma de México, Facultad de Enfermería y Obstetricia, Ciudad de México, México.; 4 Universidad de Antioquia, Facultad de Medicina, Medellín, Colombia.; 5 Hospital Pablo Tobón Uribe, Medellín, Colombia.; 6 Hospital General de México, Ciudad de México, México.; 7 Instituto Nacional de Rehabilitación Luis Guillermo Ibarra Ibarra, Ciudad de México, México.

**Keywords:** Obesity, Bariatric Surgery, Life Change Events, Weight Bias, Qualitative Research, Obesidade, Cirurgia Bariátrica, Trajetória de vida, Preconceito em relação ao peso, Pesquisa qualitativa

## Abstract

**Objective:**

To analyze the life trajectories of women who have lived with obesity since childhood and decide to undergo bariatric surgery.

**Method:**

A descriptive-exploratory qualitative study. Semi-structured interviews and photobiographies were conducted with 21 women who had undergone bariatric surgery; grounded theory analysis techniques were employed. Approval was obtained from the ethics and research committees.

**Results:**

The participants had been obese since childhood and experienced a steady increase in weight, reaching their peak during pregnancy, the loss of a loved one, or isolation due to COVID-19. They shared experiences of violence, discrimination, and stigma; they used food and isolation as a coping mechanism and a form of protection. They employed multiple strategies to lose weight, but weight regain was always greater. Limitations in performing activities of daily living led them to seek medical care, where bariatric surgery was presented as an opportunity to regain their health.

**Conclusion:**

The life trajectory of those living with obesity from an early age unfolds against a backdrop of violence; isolation, emotional eating, and situational crises exacerbate weight gain, affecting their health and leading them to bariatric surgery.

## RESUMO

**Objetivo:**

Analisar a trajetória de mulheres que vivem com obesidade desde a infância e decidem se submeter à cirurgia bariátrica.

**Método:**

Estudo qualitativo descritivo-exploratório. Foram realizadas entrevistas semiestruturadas e fotobiografias com 21 mulheres que passaram por cirurgia bariátrica, utilizando-se técnicas de análise da teoria fundamentada. Obteve-se a aprovação dos comitês de ética e pesquisa.

**Resultados:**

As participantes apresentavam obesidade desde a infância e mantiveram um aumento constante de peso, atingindo seu pico durante a gravidez, a perda de um ente querido ou o isolamento devido à COVID-19. Elas compartilharam experiências de violência, discriminação e estigma; utilizaram a comida e o isolamento como alívio e proteção. Empregaram múltiplas estratégias para perder peso, mas o ganho de peso subsequente sempre foi maior. As limitações para realizar atividades da vida cotidiana as levaram a procurar um médico, que lhes apresentou a cirurgia bariátrica como uma oportunidade para recuperar a saúde.

**Conclusão:**

A trajetória de quem vive com obesidade desde a infância ocorre em um contexto de violência; o isolamento, a alimentação emocional e as crises situacionais exacerbam o ganho de peso, afetando sua saúde, o que as leva à cirurgia bariátrica.

## INTRODUCTION

The global prevalence of obesity has risen at an alarming rate; the World Health Organization reports that from 1990 to 2022, it doubled among adults and quadrupled among adolescents^(1)^. Furthermore, the World Obesity Federation notes that obesity affects all countries and none has managed to reduce its rates; low-income countries have reported the greatest increase in recent years, and the Americas region has the highest obesity rate, at 32% for males and 37% for females^(2)^.

In Mexico, the 2020–2023 Continuous National Health and Nutrition Survey indicates that the prevalence of obesity among those over 20 years of age is 37.1%; when broken down by sex, the percentage rises to 41% among women and falls to 33% among men^(3)^.

Obesity not only affects health but also poses a threat to the global economy because it is a risk factor for the development of noncommunicable diseases^(1,4)^, which rank among the leading causes of morbidity and mortality at the global and national levels^(5,6)^. This has led to a reorganization of health care systems and an increase in spending by health care systems, households, and society, as these conditions lead to disability, premature death, and high demand for treatment resources, as well as increased early retirement and absenteeism among the working population and decreased work performance^(6)^.

Although treatment guidelines stipulate that treatment should be comprehensive, priority is given to interventions aimed at adopting a healthy lifestyle, thereby perpetuating the belief that weight control is a personal decision dependent on an individual’s willpower, even though it has been documented that lifestyle is a direct consequence of changes resulting from modernization and globalization^(1,7,8)^.

Despite the wide variety of treatments, their effectiveness is limited; the percentage of weight loss ranges from 5–10% with behavioral interventions to 25–30% with bariatric surgery. However, maintaining weight loss is difficult, and long-term weight regain occurs even with multidisciplinary interventions^(9,10)^. Those who undergo bariatric surgery have a history of repeated weight loss and gain^(11)^; this history of failure leads to feelings of guilt, frustration, insecurity, and low self-esteem, as well as depression, uncertainty about the future, and hopelessness^(11,12,13)^.

Due to the high value placed by today’s society on physical appearance, “fatphobia” has emerged, leading to cultural, institutional, and social discrimination^(8)^. Unfortunately, the healthcare system perpetuates this stigma and discrimination, as healthcare providers find it unpleasant to treat people with obesity because they view them as lacking willpower and incapable of adhering to treatment or maintaining weight loss^(14)^; they maintain discriminatory attitudes and use condescending and disrespectful language, which leads to delays in or avoidance of medical treatment^(15)^. Furthermore, public health campaigns resort to stigmatization and emphasize the individual’s responsibility for the development of obesity^(16)^. In addition, healthcare institutions lack material resources adapted to the needs of people with obesity^(14)^.

Patients have recognized these issues and report feelings of guilt and dehumanization in medical care, as any health problem is attributed to their excess weight, leading them to reduce, delay, or avoid contact with healthcare providers^(14)^.

Although obesity represents a critical challenge to public health, the traditional clinical approach often downplays the biographical dimension of individuals living with obesity, leaving a knowledge gap regarding how the lived experience unfolds—from the normalization of excess weight in childhood to the biopsychosocial breakdown that motivates the decision to undergo surgical intervention. Addressing this gap requires giving a voice to the protagonists; therefore, the objective of this research was to analyze the life trajectories of women who have lived with obesity since childhood and decide to undergo bariatric surgery.

## METHOD

### Study Design

A descriptive qualitative study using a constructivist grounded theory data analysis technique that acknowledges the participation of both the researcher and the participants in the co-construction of meanings mediated by intersubjectivity and culture^(17)^. Methodological rigor and the quality of the report were ensured through compliance with the COREQ guidelines^(18)^.

### Study Site

The study was conducted at a clinic specializing in obesity at a public hospital in Mexico City, where, in addition to providing health services, the hospital conducts training for healthcare professionals and carries out research.

The clinic provides free care to the country’s population without health insurance and charges fees to beneficiaries of other health institutions. It offers multidisciplinary care provided by staff in psychology, internal medicine, nutrition, and bariatric medicine.

### Selection Criteria and Sampling Method

Participants were personally invited by the principal investigator at the conclusion of their follow-up consultation and after a review of their medical records to confirm that they met the following inclusion criteria: being a woman over 18 years of age who had undergone bariatric surgery between 1 and 2 years prior; the exclusion criteria were: declining the invitation, not speaking Spanish, or not identifying as a woman based on their sex assigned at birth. Initially, purposive sampling was used, followed by theoretical sampling to develop the emerging categories^(17)^.

### Data Collection

Data collection was conducted by the principal investigator, a nurse with training in qualitative research. With a few exceptions, data collection took place at the clinic in a space shared with other healthcare professionals between June 2024 and February 2025. Two sessions were held with each participant, each lasting approximately 1 hour and recorded via audio. The first session consisted of a semi-structured interview that began with the trigger question: “Can you identify at what stage of your life you developed obesity?”; the second was a photobiography, which involves narrating one’s life story through the use of personal photographs, which served solely as an evocative resource. The interview script was pilot-tested with three women who had undergone bariatric surgery at a private institution; therefore, they are not part of the sample. Data collection ceased once theoretical saturation was achieved^(17)^.

### Data Analysis and Processing

Data analysis began simultaneously with data collection and was carried out in two phases: initial and focused coding^(17)^.

Open coding was performed in Word using the comments tool; infinitive verbs were used to emphasize the action; subsequently, all codes were exported to Excel, where they were grouped based on descriptive categories; a worksheet was assigned to each category to identify its properties and dimensions; finally, the central category was identified, and the information was organized around it, which allowed for the construction of the paradigmatic matrix of the phenomenon^(19)^.

The methodological rigor criteria of credibility and confirmability^(20)^ were considered to ensure credibility in the analysis of the information; four of the authors participated and continuously discussed their own interpretations and limitations. In addition, the information obtained through the two data collection techniques was triangulated, and field notes were taken on the informants’ nonverbal language and the thoughts, feelings, and sensations experienced by the principal investigator during the sessions. This was useful for reflexivity, enabling the results to be presented more objectively while remaining faithful to the participants’ experience^(21)^. With regard to confirmability, the findings are supported by the audio recordings and their verbatim transcripts. The context and conditions under which the information was collected, as well as the characteristics of the healthcare institution and the study population, were described in detail.

### Ethical Considerations

The research protocol was approved by the ethics, research, and biosafety committees of the hospital to which the clinic belongs, under registration number DI/24/401-L/03/27, which authorizes access to clinical records for the identification of potential participants.

All participants signed the informed consent form and received a copy of it, which stipulated, among other things, that their participation was voluntary and that they could withdraw from the study at any time without any repercussions; it also stipulated that the photographs taken during the second session would not be provided to the researcher by any means. To ensure the anonymity and confidentiality of the information, only the principal investigator has access to the participants’ personal data; the rest of the research team had access only to the pseudonyms—which correspond to the names of flowers—and to the transcripts of the sessions, from which all information that could reveal their identity had been previously removed.

## RESULTS

Twenty-one women between the ages of 23 and 69 participated. At the time of the interview, most were in a relationship, had children, and were employed. The surgical procedures performed were vertical sleeve gastrectomy and Roux-en-Y gastric bypass, with eight participants in each group, and five underwent mini gastric bypass. Thirteen were in their first postoperative year, and eight were in their second. Ten participants still have some degree of obesity, five are overweight, and only six have reached their normal weight.

The results are outlined in a paradigmatic matrix, which delineates the causes, context, action–interaction relationships, and consequences of the phenomenon. (Figure 1)

**Figure 1:**
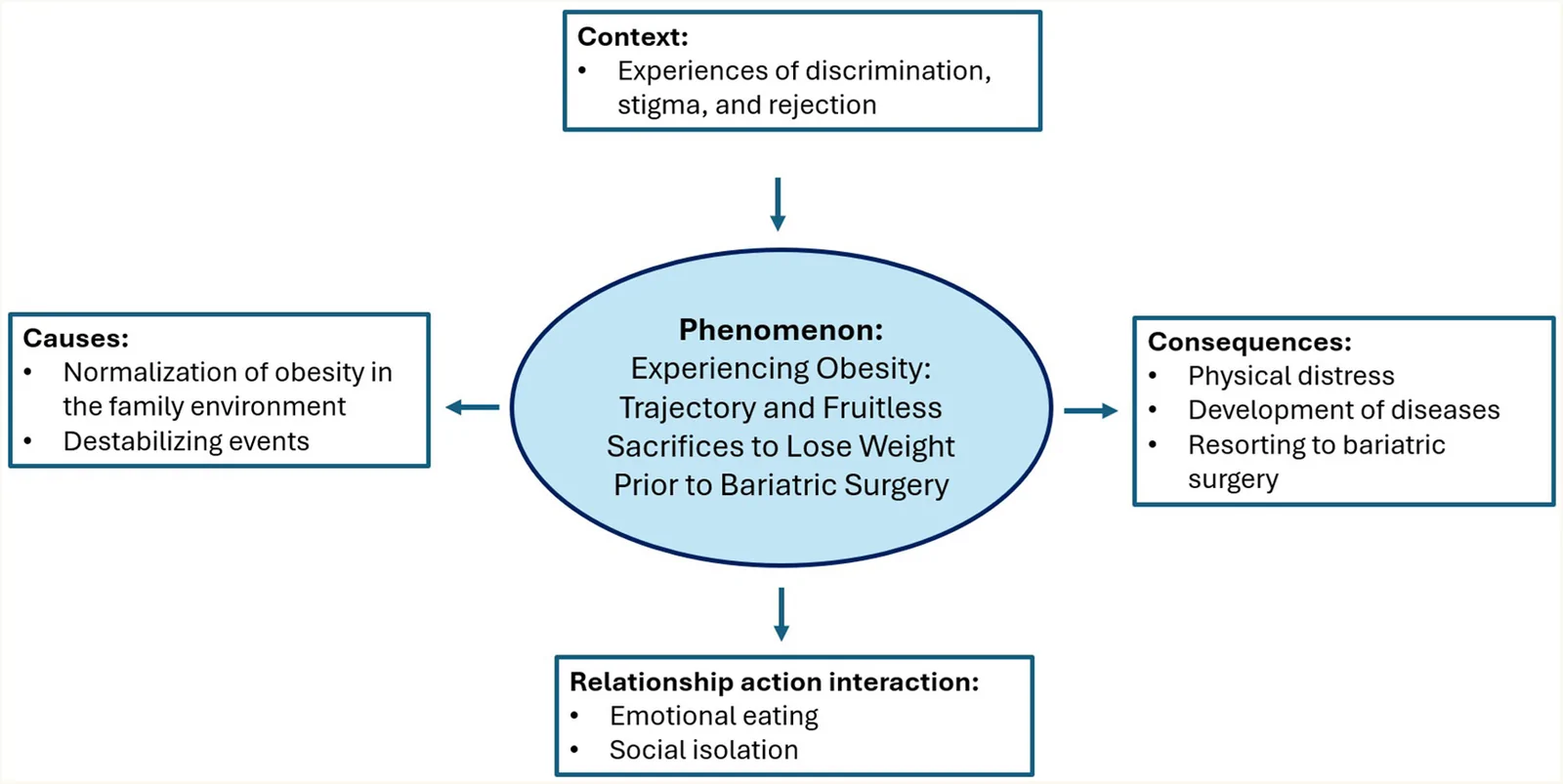
Conditional matrix of the phenomenon.

*Causes: Normalization of Obesity and destabilizing events*

For our participants, the experience of living with obesity generally begins with being overweight from childhood; however, living in a family where several members are obese leads them to normalize their situation, since to a certain extent it becomes part of their identity and sense of belonging. Some even report that, despite not feeling comfortable with their bodies, when they expressed this to their family members, they were told that they were fine just the way they were.

*Ever since we were little, all my sisters and I have been obese. My aunts were very obese—not just 140 kilos, but I think around 160, because they were huge, huge. My mom had one belly after another, all the way down to her legs. Rosa*

*They’d tell me, “You don’t look that chubby.” They always focused more on my sister’s weight; mine was nothing compared to hers […] I was the only one who noticed that I was chubby. Gardenia*

Although the participants were already overweight or obese at a young age, there were factors that led to a sudden weight gain in some of them, including, as they mention, adolescence; the arrival of children; the onset of certain illnesses; the loss of a loved one; a change of residence; and, more recently, the social isolation resulting from the pandemic. They describe these events as destabilizing, as they experienced changes in their daily routines and felt sadness, fear, anxiety, and depression.

They also agree that during this time they were focused on coping with the situation and taking care of their families, so they neglected themselves—to such an extent that they can’t pinpoint exactly when they reached their highest weight.

*“My dad passed away, and after that the twins were born. My life was a total mess, and well, I didn’t realize when it happened […] I always say, ‘One day I fell asleep, woke up, and I was already like this.’” Violeta*

The sustained weight gain among the participants stems from the normalization of childhood obesity—which provided a sense of family identity—and from destabilizing events that acted as critical triggers, making weight gain inevitable.

*Phenomenon: Experiencing Obesity: Trajectory and Fruitless Sacrifices to Lose Weight Prior to Bariatric Surgery*

The participants agree that the pressure to lose weight was always present, but during childhood, the responsibility for weight loss fell on their mothers, whom society demanded take action on their behalf.

*My dad’s family always discriminated against me because I was chubby as a child. They would ask my mom why she wasn’t doing anything for me, saying I was already too fat and should just eat less. Hortensia*

*I was in fifth grade when I went on my first diet. My mom took me to a nutritionist, but he started giving me pills derived from amphetamines. Flor de Loto*

When the participants reach adulthood, they take control of their lives. They live in a constant struggle and turn to anything that promises weight loss—sometimes guided by doctors, but mainly supported and advised by other people with obesity, since only they know what they’re talking about. When they don’t get the expected results quickly, they abandon the strategy, and the rebound sets in.

*I tried many things—I went to an allopathic doctor, a homeopath; I took all sorts of things—“take this, do that, rub this on”; I tried the sun diet, the moon diet, the stars diet—you name it. […] I took Orlistat and other medications—there were so many that I can’t even remember all their names anymore. […] I exercised […] Orlistat causes terrible diarrhea; it makes you feel awful. It was prescribed by a doctor, but I think the recommendation was a bit careless. I think that if you knew the side effects of certain treatments, you’d never do them, but you’re so desperate that you end up doing all sorts of things—sometimes unbelievable ones—that now, when I think about it, I say, “What was I thinking?” You might say, “I’m ignorant,” but no […] stopping eating, obviously. And the rebound was brutal. After that, it was brutal. Gladiola*

Some participants reported that, upon failing to lose weight, they began implementing strategies to conceal their excess weight; they used girdles to improve their appearance, and two others mentioned having resorted to cosmetic surgery, since appearance was more important to them at that moment than health.

*I always chose to wear loose-fitting black clothes, and I tried to cover myself with my posture—I’d cross my legs or hold my bag in front of me. Flor de Loto*

*For me, it had already become a really big deal; I was frustrated, so frustrated. I wore girdles all the time—real ones with underwires and little clasps—and it was torture to feel the girdle cutting off my circulation, but I wouldn’t take it off. I’d say, “No, no, because I deserve it.” Tulip*

*When my daughter took me to get a lipectomy, they gave me breast implants, performed liposuction, lifted my buttocks, and did a nose job. Yes, the whole process was very painful, but I’d do it all over again. Amarilis*

The participants’ trajectories reveal that, in the face of failed weight-loss strategies, the goal is replaced by managing one’s appearance—which highlights that, for those living with obesity, the body becomes an object that must be hidden, stylized, or altered to meet social expectations.

*Context: Experiences of Discrimination, Stigma, and Rejection*

Unlike other illnesses, obesity is visually apparent, and in a society that glorifies thinness, those who fall outside the norm experience various forms of violence, even within their own families. The persistence of these experiences leads them to feel worthless.

*Ever since I was a little girl, they called me “the fat one” or “the chubby one,” and that traumatized me; it made me a very isolated child—I couldn’t fully develop. […] In my family, the women are generally quite plump, but the men are thin, and they’d say, “Oh! Another pig in the family”—that was violence, too. Flor de Loto*

As a result of experiences associated with obesity, the participants develop a negative self-concept and devalue themselves. Some feel the need to belong to a group, so they adopt a more friendly personality; others tolerate destructive romantic relationships because they believe no one else will love them.

*I used to scold myself: “You damn fatso, keep eating—look at yourself, look at how you look.” Dalia*

*I had a lot of serious problems with my husband—infidelity and so on […] but I understand him, because if the roles were reversed, maybe I, too, would have been put off by someone who wasn’t put together, because honestly, I didn’t look good—I’d really let myself go. In fact, I have some photos that are shocking. Violeta*

By failing to meet hegemonic beauty standards, the participants adopt a complacent and submissive personality with the intention of facilitating their interpersonal relationships, thereby normalizing violence.

*Action–Interaction Relationships: Emotional Eating and Social Isolation*

In addition to these experiences, public spaces and clothing aren’t designed for their body sizes, so they take refuge in isolation as a defense mechanism, and food serves as a coping mechanism to manage the feelings they experience—which turns into a vicious cycle.

*I used to say, “If I have it, I’ll buy it; if I can buy it, I’ll eat it,” but the truth is that it was just a fantasy—my problem was that I’d go to the malls and couldn’t find my size. But the food didn’t come in different sizes; I could buy that, though. Gerbera*

*I felt like a person with a disability when there were no ramps; on the plane, the seatbelt wouldn’t fit me, so I had to tell the flight attendant, and she said, “Oh, I’ll lend you an extension” […] I have to put two or three chairs together so it doesn’t break. Azucena.*

*I started to withdraw; I began losing a lot of friends because I didn’t want to go out anywhere—I didn’t feel comfortable. I missed out on my party years because I shut myself away. I shut myself away at home to eat and be alone. Lirio*

Public spaces and clothing aren’t designed for their body sizes, so they take refuge in isolation as a defense mechanism, and once again, food emerges as a coping mechanism to manage the feelings they experience—which becomes a vicious cycle that perpetuates weight gain.

*Consequences: Physical discomfort and illness, resorting to bariatric surgery*

Over time, the problem is no longer just aesthetic; physical discomfort and illnesses begin to appear that limit the participants’ daily activities, so their family members and loved ones became concerned and intervened to encourage them to seek healthcare services.

*Physically and health-wise, I was already in very bad shape […] To be honest, I didn’t want anything anymore; I’d tell my parents, “Just leave me alone—let whatever’s going to happen to me happen,” but my brother was the one who made me see reason, and I accepted the help because I knew that no matter how much I dieted or exercised, I wasn’t going to be able to lose that weight, and my health was already at its limit.* Hortensia

*My daughter was also really scared whenever I got sick; whenever she saw me, she’d get scared. […] It was already very hard to live like that; my life was already very, very, very bad. Always feeling sick or having to go to the doctor all the time, constantly thinking, “I’m going to have a heart attack, or tachycardia, or something.” Not even being able to go out without my husband asking me, “Did you bring your insurance card?”—that’s how bad it had gotten. Narciso*

The meeting with the healthcare team is crucial, since that’s when their treating doctors suggest they undergo bariatric surgery and, in some cases, refer them to the obesity clinic.

*Two years ago, they removed my uterus, and the doctor told me, “To be honest, you’re very overweight—it was a lot of work for us; your extra weight was getting in the way.” […] Two months later, they removed my gallbladder, and I told the doctor, “If you can trim off a little fat, I won’t get mad—just slice it off.” The doctor really encouraged me and said, “If you really commit to your surgery, I’ll refer you to plastic surgery.” […] I went to the plastic surgeon, and he told me, “You need to do more—you have to lose weight first so we can remove that sagging skin. I’m going to refer you to bariatric surgery.” Margarita*

*I had a lot of problems with reflux, heartburn, and a hiatal hernia. I was treated by a gastroenterologist for many years until I went to the Upper Digestive Tract clinic. There, they examined me, sent me for an endoscopy, and told me, “You’re in very bad shape—this is already very advanced. You’re still in time for surgery.” That’s when the doctor mentioned the bypass. Dalia*

In general, the participants learned about bariatric surgery through referrals from their doctors; however, some discovered it through television, social media, or acquaintances who had undergone the procedure themselves. Seeing positive results in a short period of time leads them to view surgery as a possibility. Some begin their journey toward surgery directly at the obesity clinic, accompanied by other patients who guide them through the process, while other participants first go to a private facility where the cost of surgery is high, so they decide to seek care at a public hospital.

*My daughter’s friend used to be like a little ball—I mean, really short, really short, but chubby, chubby, chubby, chubby—and when I saw her again, she was all slim, and I was surprised. My daughter told me, “Mom, I was talking to her about her surgery, and she told me they did it at the hospital and they only charged her the copay, and she just bought her medications. […] And like they say, she guided me—she paved the way for me.” Amarilis*

*A cousin of mine had the surgery and showed us how much weight she’d lost and everything, and the idea of getting the surgery started to take root in my mind. On Facebook, I came across a doctor offering a free consultation, so I made an appointment with him in Mexico. The two of us—my husband and I—went, but when he told us how much it would cost, I said, “I might as well die fat because I don’t have that kind of money”; we were just coming out of the pandemic, having lost our jobs, and were just starting to get back on our feet. […] My cousin, who’s a doctor, suggested I go to the obesity clinic, so I went through all the paperwork, went to sign up, and made an appointment—and to my surprise, the doctor was the same one I’d seen at the private clinic. Azucena*

Excess weight ultimately undermines health, leading to chronic degenerative diseases and severe limitations in performing activities of daily living, which drives patients to seek professional help. Bariatric surgery is often seen as the ideal treatment option; however, in private institutions, it is not accessible to the entire population, so patients turn to public health services, where the surgery is free.

## DISCUSSION

The meaning that participants ascribe to obesity changes over the course of their lives, evolving from something “normal,” to a problem with body image, and finally to a disease. This aligns with the findings of a study conducted among the Mexican population, which concluded that obesity is perceived as a health problem only in stages II and III, since recognizing it as a disease implies experiencing discomfort, symptoms, or obesity-related conditions—which could delay the search for care^(22)^.

Throughout their lives, women with obesity experienced situations of violence, beginning within their immediate families and later in all areas of their lives; some authors emphasize that it is essential to recognize obesity as a disease so that the responsibility for weight loss shifts from the individual to the societal level, resources are allocated for its prevention, treatment, and research, and efforts are made to reduce discrimination, blame, and the stigma surrounding treatment^(23,24)^.

Our participants employed weight-loss strategies beginning in childhood, with their mothers playing a leading role as they were recognized as responsible for their health. In this regard, the literature indicates that, although the social environment in which children grow up influences the development of obesity, it is parents who exert the closest and most decisive influence on weight-related behaviors^(25)^, nevertheless, the responsibility falls on the mother, as she is the one in charge of the family’s diet^(26)^.

With a few exceptions where doctors prescribed medication, the participants agree that healthcare treatment focuses on diet and exercise, which reveals that, although scientific evidence advocates for comprehensive and multimodal treatments, priority is given to interventions aimed at lifestyle modification, sometimes with the support of cognitive-behavioral therapy^(27)^, such treatment is less effective than other options, such as pharmacological and/or surgical interventions^(10)^, and it also perpetuates the belief that weight control is a personal decision.

The participants used various strategies to lose weight; however, there was always significant weight regain. The literature reports that some people tend to hide these experiences for fear of being judged and/or blamed^(28)^; however, this was not the case in our study. On the contrary, they often discussed their strategies and recommended them to one another. With weight regain came illnesses and/or physical discomfort, leading the participants to seek healthcare services where bariatric surgery was proposed as a treatment. It is worth noting that, although most were referred to the bariatric service by their doctors, some learned about the surgery through acquaintances or the media, which differs from the findings of another study stating that the majority of patients learn about bariatric surgery through social media (37.3%) compared to 27.3% who learn about it through their primary care physician^(29)^.

Finally, our findings reaffirm that individuals living with obesity who opt for bariatric surgery tend to construct narratives that justify their weight gain and validate the surgical procedure as a means to improve their health—rather than as an easy way out or a consequence of personal failure—since these narratives help them build a coherent and respectable identity; Furthermore, they attribute weight gain to individual factors or life events that prevent them from adopting a healthy lifestyle, without considering the influence of the obesogenic environment and/or social determinants^(30)^—which suggests an internalization of the belief that obesity is the responsibility of the person who suffers from it.

One limitation was the lack of a private space for data collection, which at times made them hesitant to speak openly about their deepest emotions.

## CONCLUSION

The life trajectories of women who have lived with obesity since early childhood are shaped by a context of discrimination, stigma, and rejection, where isolation and emotional eating emerge as coping strategies that, over time, exacerbate excess weight and negatively impact the biopsychosocial health of those affected.

Some strategies, such as wearing a compression garment or undergoing plastic surgery, reveal that obesity is initially recognized as an aesthetic problem, where the focus is not on resolving the health issue but on improving physical appearance to live a fuller life; therefore, bariatric surgery represents an opportunity not only to improve health but also aligns with their desire to drastically transform their physical appearance in a short period of time.

Contributions to the discipline: Recognizing this phenomenon allows nursing professionals to understand obesity from the perspective of those who suffer from it, which contributes to the development of person-centered care interventions.

## Data Availability

The entire dataset supporting the results of this study was published in the article itself.
