# Supplementary material for: Experiencing obesity: trajectory and fruitless sacrifices to lose weight prior to bariatric surgery
Source: Rev Esc Enferm USP. 2026 Sep 25;60:e20260209. doi: 10.1590/1980-220X-REEUSP-2026-0209en (PMC13619143; doi:10.1590/1980-220X-REEUSP-2026-0209en)
Supplement: Translation

# Translation: Vivir la obesidad: Trayectoria y sacrificios infructuosos para perder peso previo a la cirugía bariátrica

**DOI:** 10.1590/1980-220X-REEUSP-2026-0209es

**Authors:** Blessing Itzel Palacios-Meneses, Sarah Bak-Geller Corona, Araceli Jiménez-Mendoza, Carlos Enrique Yepes Delgado, Antonio González Chavez, Laura Olivia Pech Rueda, Sandra Hernández-Corral

**Affiliations:** 1 Universidad Nacional Autónoma de México, Facultad de Estudios Superiores Iztacala, Estado de México, México.; 2 Universidad Nacional Autónoma de México, Instituto de Investigaciones Antropológicas, Ciudad de México, México.; 3 Universidad Nacional Autónoma de México, Facultad de Enfermería y Obstetricia, Ciudad de México, México.; 4 Universidad de Antioquia, Facultad de Medicina, Medellín, Colombia.; 5 Hospital Pablo Tobón Uribe, Medellín, Colombia.; 6 Hospital General de México, Ciudad de México, México.; 7 Instituto Nacional de Rehabilitación Luis Guillermo Ibarra Ibarra, Ciudad de México, México.

**Keywords:** Obesidad, Cirugía Bariátrica, Trayectoria de vida, Sesgo de peso, Investigación cualitativa

## Abstract

**Objetivo:**

Analizar la trayectoria de mujeres que han vivido con obesidad desde la infancia y deciden realizarse cirugía bariátrica.

**Método:**

Estudio cualitativo descriptivo-exploratorio. Se realizaron entrevistas semiestructuradas y fotobiografías a 21 mujeres posoperadas de cirugía bariátrica, se emplearon técnicas de análisis de teoría fundamentada. Se obtuvo la aprobación de los comités de ética e investigación.

**Resultados:**

Las participantes presentaban obesidad desde la infancia y mantuvieron un incremento constante de peso llegando a su máximo con el embarazo, la pérdida de un ser querido o el aislamiento por COVID-19. Comparten experiencias de violencia, discriminación y estigma; utilizan la comida y aislamiento como paliativo y protección. Emplearon múltiples estrategias para perder peso, la reganancia siempre fue mayor. Las limitaciones para realizar actividades de la vida cotidiana hicieron que acudieran al médico quien les planteó la cirugía bariátrica como una oportunidad para recuperar su salud.

**Conclusión:**

La trayectoria de quienes viven con obesidad desde edades tempranas se da en un contexto de violencia; el aislamiento, comer emocional y crisis situacionales exacerban el aumento de peso afectando su salud lo que las lleva a la cirugía bariátrica.

## INTRODUCCIÓN

La prevalencia mundial de obesidad ha tenido un incremento alarmante, la Organización Mundial de la Salud reporta que de 1990 a 2022 se duplicó en adultos y cuadriplicó en adolescentes^(1)^, por otro lado, la Federación Mundial de la Obesidad menciona que la obesidad afecta a todos los países y ninguno ha logrado disminuir sus cifras; los de más bajos ingresos son lo que reportan un incremento mayor en los últimos años y la región de las Américas es la que presenta la proporción más alta de obesidad con 32% para el sexo masculino y 37% para el sexo femenino^(2)^.

En México la Encuesta Nacional de Salud y Nutrición Continua 2020-2023 menciona que la prevalencia en obesidad en mayores de 20 años es del 37.1%, y al estadificarla por sexo el porcentaje incrementa al 41% en mujeres y disminuye al 33% en hombres^(3)^.

La obesidad no solo repercute en la salud sino que también representa una amenaza para la economía mundial debido a que representa un factor de riesgo para el desarrollo de las Enfermedades No Transmisibles^(1,4)^, las cuales se ubican entre las principales causas de morbilidad y mortalidad a nivel mundial y nacional^(5,6)^ lo que ha implicado una reorganización de los sistemas de salud y un aumento en el gasto de los sistemas sanitarios, los hogares y la sociedad, debido a que traen consigo discapacidad, muerte prematura y una alta demanda de recursos para su tratamiento, así como un incremento de abandono prematuro y ausentismo laboral de la población activa y disminución del rendimiento laboral^(6)^.

A pesar de que las guías de tratamiento establecen que el tratamiento debe ser integral, se priorizan las intervenciones encaminadas a la adopción de un estilo de vida saludable con lo cual se perpetua la creencia de que el control de peso es una decisión personal que depende de la voluntad de la persona, aun cuando se ha documentado que el estilo de vida es consecuencia directa de los cambios derivados de la modernización y globalización^(1,7,8)^.

A pesar de la amplia variedad de tratamientos la efectividad de estos es reducida, el porcentaje de pérdida de peso oscila de 5–10% con intervenciones conductuales hasta un 25–30% con cirugía bariátrica, no obstante, mantener la pérdida de peso es complicado y existe reganancia de peso a largo plazo aun con intervenciones multidisciplinarias^(9,10)^. Quienes se someten a cirugía bariátrica tienen un historial repetido de pérdidas y aumentos de peso^(11)^ dicho historial de fracasos provoca sentimientos de culpa, frustración, inseguridad y desconfianza en sí mismas, así como depresión, incertidumbre hacia el futuro y desesperanza^(11,12,13)^.

Debido a que en la sociedad actual se rinde culto al cuerpo ha aparecido la “gordofobia” la cual conlleva a la discriminación cultural, institucional y social^(8)^. Desafortunadamente en el sistema de salud se replica el estigma y discriminación ya que se percibe como desagradable tratar con personas que presentan obesidad debido a que las identifican como carentes de fuerza de voluntad e incapaces de adherirse al tratamiento o mantener la pérdida de peso^(14)^, mantienen actitudes discriminatorias y emplean un lenguaje condescendiente e irrespetuoso, lo que conlleva al retraso o evitación del tratamiento médico^(15)^. Por otro lado, las campañas de salud pública recurren a la estigmatización y enfatizan la responsabilidad de la persona para el desarrollo de la obesidad^(16)^. Aunado a esto, dentro de las instituciones faltan recursos materiales que se adapten a la persona con obesidad^(14)^.

Lo anterior expuesto ha sido percibido por los pacientes, quienes manifiestan sentimientos de culpa y deshumanización en la atención médica, ya que cualquier problema de salud es atribuido a su exceso de peso, lo cual conlleva a una reducción, retraso o evitación de contacto con el equipo de salud^(14)^.

Si bien la obesidad representa un desafío crítico para la salud pública, el enfoque clínico tradicional suele minimizar la dimensión biográfica de la persona que padece obesidad, dejando un vacío del conocimiento sobre cómo se articula el proceso vivido desde la normalización del exceso de peso en la infancia hasta el colapso biopsicosocial que motiva la decisión de someterse a una intervención quirúrgica. Abordar este vacío exige dar voz de las protagonistas, por lo que el objetivo de esta investigación fue analizar la trayectoria de mujeres que han vivido con obesidad desde la infancia y deciden realizarse una cirugía bariátrica.

## MÉTODO

### Diseño del Estudio

Estudio cualitativo descriptivo con técnica de análisis de datos de teoría fundamentada constructivista que reconoce la participación del investigador y los participantes en la co-construcción de los significados que se encuentran mediados por la intersubjetividad y la cultura^(17)^. El rigor metodológico y la calidad del reporte se garantizaron mediante el cumplimiento de la guía COREQ^(18)^.

### Local del Estudio

El estudio se llevó a cabo en una clínica especializada en obesidad de un hospital público de la Ciudad de México en el que además de la prestación de servicios de salud se lleva a cabo la formación de recursos humanos en salud y el desarrollo de investigación.

Dentro de la clínica se proporciona atención gratuita a la población sin seguridad social del país y con costo a derechohabientes de otras instituciones de salud. Ofrece atención multidisciplinaria integrada por personal de psicología, medicina interna, nutrición y bariatría.

### Criterios de Selección y Tipo de Muestreo

Las participantes fueron invitadas personalmente por la investigadora principal al culminar su consulta de seguimiento y revisión del expediente clínico para corroborar que cumpliera con los siguientes criterios de inclusión: ser mujer mayor de 18 años y tener entre 1 y 2 años de posoperada de cirugía bariátrica; los criterios de exclusión fueron: la declinación a dicha invitación, que no hablaran español o no sean mujeres por su sexo asignado al nacer. Inicialmente se empleó muestreo intencional y posteriormente se procedió a un muestreo teórico para el desarrollo de las categorías emergentes^(17)^.

### Recolección de Datos

La recolección de datos estuvo a cargo de la investigadora principal, quien es enfermera, con formación en investigación cualitativa. Salvo algunas excepciones se realizó dentro de la clínica en un espacio compartido con otros profesionales de la salud entre junio 2024 y febrero 2025. Se tuvieron 2 encuentros con cada participante que tuvieron una duración aproximada de 1 hora y fueron grabados en audio. El primer encuentro consistió en una entrevista semiestructurada que partió de la pregunta detonadora ¿Logra identificar en qué etapa de su vida desarrolló obesidad?; el segundo fue una fotobiografía, la cual consiste en la narración de la historia de vida a través del uso de fotografías personales, que fungieron únicamente como un recurso evocativo. El guion de entrevista fue piloteado en 3 mujeres posoperadas de cirugía bariátrica en una institución privada, por lo que no forman parte de la muestra. La recolección de datos continuó hasta lograr la saturación teórica^(17)^.

### Análisis y Tratamiento de los Datos

El análisis de la información fue iniciado en simultánea con la recolección de los datos y se llevó a cabo en 2 fases: codificación inicial y focalizada^(17)^.

La codificación abierta se realizó en Word a través de la herramienta de comentarios, se emplearon verbos en infinitivo para hacer énfasis en la acción; posteriormente todos los códigos se exportaron a Excel donde se agruparon con base en las categorías descriptivas, se asignó una hoja a cada una de estas para identificar sus propiedades y dimensiones, finalmente se identificó la categoría central, y la información se organizó entorno a ésta, lo que permitió la construcción de la matriz paradigmática del fenómeno^(19)^.

Se consideraron los criterios de rigor metodológico de credibilidad y confirmabilidad^(20)^ para garantizar la credibilidad en el análisis de la información participaron cuatro de los autores quienes discutieron permanentemente sus propias interpretaciones y limitaciones. Además, se trianguló la información obtenida a través de las dos técnicas de recolección de datos y se tomaron notas de campo sobre el lenguaje no verbal de las informantes y los pensamientos, sentimientos y sensaciones experimentados por la investigadora principal durante las sesiones, lo cual fue de utilidad en la reflexividad para mostrar los resultados de manera más objetiva siendo fiel a la experiencia de las participantes^(21)^. Por lo que respecta a la confirmabilidad se tiene respaldo de las grabaciones en audio y las transcripciones textuales de estas. Se describió de manera detallada el contexto y condiciones en que fue recolectada la información y las características de la institución de salud y la población de estudio.

### Aspectos Éticos

El protocolo de investigación fue aprobado por los comités de ética, investigación y bioseguridad del hospital al que pertenece la clínica con la clave de registro DI/24/401-L/03/27, el cual autoriza la consulta del expediente clínico para la identificación de potenciales participantes.

Todas las participantes firmaron el consentimiento informado y recibieron una copia del mismo, en el cual se estipuló entre otras cosas que su participación es voluntaria, y pueden retirarse de la investigación en el momento en que lo decidan sin ninguna repercusión, así mismo se estipulo que las fotografías empleadas en el segundo encuentro no se entregarían a la investigadora por ningún medio. Para garantizar el anonimato y confidencialidad de la información, únicamente la investigadora principal conoce los datos personales de las participantes, el resto de los integrantes del equipo solo tuvieron acceso al seudónimo que corresponde a nombres de flores y a las transcripciones de los encuentros, en las que previamente se eliminó toda información que pudiera revelar su identidad.

## RESULTADOS

Participaron 21 mujeres de entre 23 y 69 años. Al momento de la entrevista la mayoría mantenía una relación de pareja, tenía hijos y trabajaba. Los procedimientos quirúrgicos fueron gastrectomía vertical en manga y el bypass gástrico en Y de Roux con ocho participantes cada grupo y cinco mini bypass gástrico. Trece se encontraban en el primer año posoperatorio y ocho en el segundo año. Diez participantes aun presentan algún grado de obesidad, cinco tienen sobrepeso, solo seis llegaron a su peso normal.

Los resultados se esquematizan en una matriz paradigmática, en la que se delimitan las causas, el contexto, las relaciones de acción – interacción, y consecuencias del fenómeno. (Figura 1)

**Figura 1:** Matriz condicional del fenómeno.

*Causas: normalización de la obesidad y eventos desestabilizadores*

En nuestras participantes el fenómeno de vivir la obesidad en general comienza con la presencia de exceso de peso desde la infancia, sin embargo, el vivir en una familia donde diversos integrantes presentan obesidad las lleva a normalizar su situación, pues hasta cierto punto se convierte en parte de su identidad y pertenencia. Incluso algunas refieren que, a pesar de no sentirse cómodas con su cuerpo, cuando lo externaban con sus familiares ellos les decían que así estaban bien.

*Desde chiquitas todas mis hermanas y yo somos obesas. Mis tías eran muy obesas, obesas no de140 kilos, eran yo creo de 160, porque eran enormes, enormes. Mi mamá tenía una panza, tras otra panza llegando hasta las piernas. Rosa*

*Me decían “no te ves tan gordita” siempre se enfocaron más en el peso de mi hermana, el mío no era nada a comparación al de ella […] yo era la única que notaba que estaba gordita. Gardenia*

Si bien, las participantes ya tenían sobrepeso u obesidad en edades tempranas, existieron factores que en algunas de ellas propiciaron un incremento brusco de peso, entre ellos refieren, la etapa de adolescencia; la llegada de los hijos; el desarrollo de algunas enfermedades; la pérdida de un ser querido; el cambio de residencia y más recientemente el aislamiento social derivado de la pandemia. Ellas describen estos eventos como desestabilizadores ya que vivieron cambios en su rutina diaria y experimentaron tristeza, miedo, ansiedad y depresión.

Coinciden también en que durante esta etapa estaban enfocadas en sobrellevar la situación y cuidar de sus familias por lo que se olvidaron de sí mismas, a tal grado que les es imposible reconocer en qué momento alcanzaron su peso máximo.

*Falleció mi papá y posterior a eso los gemelos nacieron, mi vida fue un desajuste total, y pues no me di cuenta en qué momento pasó […] siempre digo “un día me dormí, desperté, y ya estaba así”. Violeta*

El incremento sostenido de peso en las participantes responde a la normalización de la obesidad en la infancia que provee de identidad familiar y a los eventos desestabilizadores que operaron como desencadenantes críticos, donde la ganancia de peso fue inevitable.

*Fenómeno: vivir la obesidad: trayectoria y sacrificios infructuosos para perder peso previo a la cirugía bariátrica*

Las participantes coinciden en que la presión por bajar de peso siempre estuvo presente, pero durante la infancia la responsabilidad de la pérdida de peso la asumían sus madres, a quienes la sociedad les exige hacer algo por ellas.

*La familia de mi papá siempre me discriminó porque desde niña siempre fui gordita. Le decían a mi mamá que por qué no hacía nada por mí, que ya estaba muy gorda que ya comiera menos. Hortensia*

*Iba en quinto de primaria cuando hice mi primera dieta. Mi mamá me llevo con un nutricionista, pero me empezó a dar pastillas derivadas de anfetaminas. Flor de Loto*

Cuando las participantes llegan a la edad adulta toman las riendas de su vida. Viven en una lucha constante y recurren a todo lo que prometa la pérdida de peso, a veces orientadas por médicos, pero principalmente acompañadas y orientadas por otra persona con obesidad pues solo ellas saben de lo que hablan. Al no obtener los resultados esperados de forma rápida abandonan la estrategia y viene el rebote.

*Hice muchas cosas, fui con un médico alópata, homeópata, tomé muchas cosas “tómate, hazte, úntate”, la dieta del sol, la luna, las estrellas, de todas. […] Tomé Orlistat y otras que hasta los nombres de tantos se me van. […] Hice ejercicio […] el Orlistat provoca unas diarreas tremendas, te hace sentirte muy mal, fue indicación médica, pero yo creo que un poco a la ligera. Pienso que si conociera uno los efectos secundarios de ciertos tratamientos no lo harías nunca, pero está uno tan desesperada que caes en hacer una cantidad de cosas a veces inverosímiles, que ahora lo pienso digo ¿qué tenía yo en la cabeza” dijeras tú, soy una ignorante, pero no […] dejar de comer, obvio. Y el rebote pues brutal. Después de eso, brutal. Gladiola*

Algunas participantes refieren que al no lograr la pérdida de peso comenzaron a implementar estrategias para disimular el sobrepeso, empleaban fajas para mejorar su apariencia y 2 más mencionan haber recurrido a cirugías estéticas, pues lo importante en este momento más que la salud era la apariencia.

*Siempre optaba por usar ropa negra y holgada y con mi postura trataba de cubrirme, cruzaba la pierna o ponía la bolsa enfrente. Flor de Loto*

*Para mí era ya una cosa muy fuerte, estaba frustrada, frustrada, me la pasaba con fajas todo el tiempo, pero con fajas de verdad de varillas y de brochecitos y era una tortura sentir que la faja te está cortando la circulación y no me la quitaba. Yo decía “no, no, porque me lo merezco. Tulipán*

*Cuando mi hija me llevó a que me hicieran la lipectomía, me pusieron chichis, me hicieron liposucción, me levantaron las nalgas y me la operaron la nariz. Sí fue muy doloroso todo ese proceso, pero lo volvería a hacer. Amarilis*

La trayectoria de las participantes revela que, ante el fracaso de las estrategias para perder peso, el objetivo es reemplazado por la gestión de la apariencia lo que pone de manifiesto que, para quienes viven con obesidad, el cuerpo se convierte en un objeto que debe ser ocultado, estilizado o intervenido para cumplir con las expectativas sociales.

*Contexto: Experiencias de discriminación, estigma y rechazo*

La obesidad a diferencia de otras enfermedades es evidente a la vista y en una sociedad en la que se enaltece el cuerpo delgado, quien se encuentra fuera del estándar experimenta diversas formas de violencia, incluso dentro de la misma familia. La persistencia de estas experiencias las lleva a desvalorizarse.

*Desde niña me decían la gorda o la cachetona y eso me traumaba, me hizo una niña muy aislada, no me pude desenvolver completamente. […] En mi familia las mujeres generalmente son muy gorditas pero los hombres son delgados y decían “¡Ay! otra marrana más en la familia”, eso era violencia también. Flor de Loto*

Derivado de las experiencias asociadas con la obesidad, las participantes desarrollan un autoconcepto negativo y se desvalorizan. Algunas, sienten la necesidad de pertenecer a un grupo, por lo que adoptan una personalidad más amigable, algunas toleran relaciones de pareja destructivas pues creen que nadie más las va a querer.

*Yo sí me regañaba “condenada tripona, sigue comiendo, mira cómo estás, mira cómo te ves” Dalia*

*Con mi esposo tuve muchos problemas fuertes, infidelidades y demás […] pero lo entiendo, porque si fuera al revés, tal vez también, me hubiera dado pena una persona desalineada, porque la verdad no me veía bien, me dejé bastante, de hecho, tengo fotos que son impactantes. Violeta*

Al no cumplir con los estándares de belleza hegemónicos, las participantes adoptan una personalidad complaciente y sumisa con la intención de facilitar sus relaciones interpersonales, normalizando la violencia.

*Relaciones de acción – interacción: comer emocional y aislamiento social*

Aunado a estas experiencias, los espacios públicos y la ropa no están diseñados para las dimensiones de su cuerpo, por lo que se refugian en el aislamiento como mecanismo de defensa y la comida aparece como un paliativo para gestionar los sentimientos que experimentan, lo cual se convierte en un círculo vicioso.

*Yo decía “si lo tengo, me lo compro, si me lo puedo comprar, me lo como” pero, la verdad es que era una fantasía, mi problema era que iba a los centros comerciales y no encontraba tallas. Pero en la comida no había tallas, eso sí lo podía comprar. Gerbera*

*Me sentía como un minusválido cuando no hay rampas; en el avión no me dio el cinturón de seguridad, le tuve que decir a la aeromoza y me dijo “Ah, le presto una extensión” […] tengo que poner 2 o 3 sillas para que no se vaya a romper. Azucena.*

*Me empecé a alejar; empecé a perder muchas amistades porque yo no quería salir a ningún lado, no me sentía cómoda. No viví mi etapa de fiestas porque me encerré. Me encerré en mi casa a comer y a estar sola. Lirio*

Los espacios públicos y la ropa no están diseñados para las dimensiones de su cuerpo, por lo que se refugian en el aislamiento como mecanismo de defensa y nuevamente la comida aparece como un paliativo para gestionar los sentimientos que experimentan, lo cual se vuelve un círculo vicioso que perpetua el incremento de peso.

*Consecuencias: malestar físico y enfermedades, recurrir a la cirugía bariátrica*

Con el paso del tiempo el problema ya no es sólo estético, sino que comienzan a aparecer malestares físicos y enfermedades que limitan las actividades de la vida diaria de las participantes, por lo que sus familiares y seres queridos se preocupaban por ellas e intervinieron para que se acercaran a los servicios de salud.

*Físicamente y de salud ya estaba muy mal […] Para ser sincera ya no quería nada, les decía a mis papás “déjenme ya, que me pase lo que me tenga que pasar” pero mi hermano fue el que me hizo entrar en razón y acepté la ayuda porque yo sabía que ni con dieta, ni por más ejercicio que hiciera, iba a lograr bajar ese peso y mi salud ya estaba en el límite.* Hortensia

*Mi hija también ya se espantaba mucho cuando me ponía mal, las veces que me llegaba a ver se espantaba. […] Ya era muy difícil vivir así, mi vida ya era muy, muy, muy mala. Vivir siempre sintiéndome mal o tener que acudir al doctor todo el tiempo, estar pensando “ya me va a dar algo, no sé un infarto, una taquicardia, o algo” No poder ni siquiera salir sin que mi esposo me dijera “¿Ya traes tu carnet?” a ese grado llegué. Narciso*

El encuentro con el equipo sanitario resulta crucial, pues es ahí donde sus médicos tratantes les sugieren se realicen una cirugía bariátrica y en algunos casos las derivan a la clínica de obesidad.

*Hace 2 años, me quitaron la matriz y el doctor me dijo” la verdad, tienes mucho sobrepeso nos costó mucho trabajo, nos estorbaba tu gordito” […] A los 2 meses me retiran la vesícula y le dije a la doctora, “si me puedes cortar un poquito de grasa, yo no me enojo usted rebáaanele” La doctora me motivó mucho y me dijo, “si tú le echas ganas a tu cirugía, te voy a mandar a cirugía plástica”. […] Voy a la cirugía plástica y el doctor me dice “ te falta más, necesitas primero bajar de peso, para que esa piel que se te cuelgue la podamos eliminar. Te voy a mandar a bariatría” Margarita*

*Tenía muchos problemas de reflujo, de acidez y una hernia hiatal, me atendí en gastro por muchos años hasta que llegué a Tracto Digestivo Superior, ahí me atendieron, me mandaron a hacer la endoscopia y me dice “estás muy mal, muy mal, esto ya está muy avanzado, estás a tiempo de una cirugía”, ahí me comentó el doctor del bypass. Dalia*

En general las participantes conocieron sobre la cirugía bariátrica por referencia de sus médicos, sin embargo, algunas la descubrieron a través de la televisión, redes sociales o por conocidos que comparten el haberse realizado dicha intervención. El ver resultados favorables en un periodo corto de tiempo las lleva a visualizar la cirugía como una posibilidad. Algunas comienzan su búsqueda hacía la cirugía directamente en la clínica de obesidad, acompañadas de otras pacientes quienes las orientan en los tramites, mientras que otras participantes acuden primero a una institución privada donde el costo de la cirugía es elevado, por lo que deciden buscar atención en un hospital público.

*Su comadre de mi hija era igual una pelotita, o sea, chaparrita, chaparrita, pero gordita, gordita, gordita, gordita, cuando la vuelvo a ver la veo delgadita y me sorprendí. Me dice mi hija “mamá, estaba hablando con ella de su cirugía y me dice que se la hicieron en el hospital y que nomás le cobraron lo de su carnet y compró medicamentos. […] Y como dicen por ahí, ella me guio, me abrió el camino. Amarilis*

*Se opera un primo mío y nos enseña cómo ha bajado y todo y se me empieza a meter en la cabeza esa idea de operarme. En Facebook me sale un doctor con valoración gratis e hice la cita con él en México, fuimos los 2, mi marido y yo, pero cuando nos dijo cuánto cobraban dije “ya me morí gorda porque no tengo”; veníamos de pandemia, de perder trabajos, de estarnos levantando. […] Mi prima que es doctora me sugirió acudir a la clínica de obesidad, entonces hice todo mi trámite, me fui a formar y pedí cita y ¿cuál es mi sorpresa? el doctor es con el que yo había ido en el particular. Azucena*

El exceso de peso termina por deteriorar la salud detonando en enfermedades crónico-degenerativas y limitaciones severas para la realización las actividades de la vida diaria, lo que orilla a las pacientes a buscar ayuda profesional, donde la cirugía bariátrica se vislumbra como la opción de tratamiento ideal, sin embargo, en instituciones privadas no está al alcance de toda la población, por lo que recurren a los servicios públicos de salud, donde la cirugía es gratuita.

## DISCUSIÓN

El significado que las participantes otorgan a la obesidad se va transformando a lo largo de su vida pasando de ser algo “normal”, a un problema de apariencia corporal y finalmente una enfermedad, lo que coincide con lo reportado en un estudio realizado en población mexicana que concluye que la obesidad se percibe como problema de salud únicamente en los estadios II y III ya que reconocerla como enfermedad implica presentar molestias, síntomas o enfermedades asociadas a la obesidad lo que podría retrasar la búsqueda de atención^(22)^.

A lo largo de su vida, las mujeres con obesidad vivieron situaciones de violencia, desde su núcleo familiar y posteriormente en todos los ámbitos de su vida; algunos autores resaltan que, es indispensable reconocer a la obesidad como una enfermedad para que la responsabilidad de la pérdida de peso se traslade de lo individual a lo social y se gestionen recursos para su prevención, tratamiento e investigación; y se contribuya a la reducción de la discriminación, culpabilización y el estigma del tratamiento^(23,24)^.

Nuestras participantes hicieron uso de estrategias para perder peso desde la infancia donde sus madres tenían el rol protagónico al ser reconocidas como las responsables de su salud, referente a esto la literatura señala que, si bien el entorno social en que crecen los niños influye en el desarrollo de la obesidad, son los padres quienes ejercen la influencia más cercana y determinante sobre las conductas relacionas con el peso^(25)^, no obstante la responsabilidad recae sobre la madre pues es quien se encarga de la alimentación familiar^(26)^.

Salvo algunas excepciones donde los médicos prescribieron fármacos, las participantes coinciden en que en la atención sanitaria el tratamiento se centra en la dieta y ejercicio, lo cual deja ver que, a pesar de que la evidencia científica aboga por tratamientos integrales y multimodales, se priorizan las intervenciones encaminadas a la modificación del estilo de vida, en ocasiones con el apoyo de la terapia cognitivo conductual^(27)^, dicho tratamiento tiene una eficacia menor frente a otras opciones como el farmacológico y/o quirúrgico^(10)^, además de perpetuar la creencia de que el control de peso es una decisión personal.

Las participantes emplearon diversas estrategias para perder peso, sin embargo, siempre había una reganancia mayor; la literatura reporta que algunas personas suelen ocultar dichas experiencias por temor a ser juzgadas y/o culpabilizadas^(28)^, sin embargo, en nuestro estudio esto no se manifestó, al contrario, solían comentar sus estrategias y recomendarlas entre ellas. Con la reganancia de peso llegaron las enfermedades y/o malestares físicos por lo que las participantes recurrieron a los servicios de salud donde la cirugía bariátrica les fue propuesta como tratamiento. Cabe resaltar que, si bien la mayoría fueron referidas por sus médicos al servicio de bariatría, algunas conocieron la cirugía a través de conocidos o medios de comunicación, lo cual difiere a lo reportado en otro estudio que afirma que el mayor porcentaje de sus pacientes se enteran de la cirugía bariátrica a través de redes sociales (37.3%) frente al 27.3% que la conocen por medio de su médico de cabecera^(29)^.

Finalmente, nuestros hallazgos reafirman que quienes viven con obesidad y optan por la cirugía bariátrica, tienden a establecer narrativas que justifiquen su incremento de peso y validen la intervención quirúrgica como un recurso para mejorar su salud y no como la salida fácil o consecuencia de su fracaso personal, ya que con estas narrativas construyen una identidad coherente y respetable; además atribuyen el incremento de peso a factores individuales o acontecimientos de la vida que impiden la adopción un estilo de vida saludable sin considerar la influencia del ambiente obesogénico y/o determinantes sociales^(30)^ lo que nos habla de una introyección de las creencias de que la obesidad es responsabilidad de quien lo padece.

Una limitante fue no disponer de un espacio privado para la recolección de datos lo que en ocasiones las cohibía para hablar abiertamente sobre sus emociones más profundas.

## CONCLUSIÓN

La trayectoria de las mujeres que viven con obesidad desde edades tempranas se enmarca en un contexto de discriminación, estigma y rechazo donde el aislamiento y comer emocional surgen como estrategia de afrontamiento que con el tiempo exacerban el exceso de peso e impactan negativamente en la salud biopsicosocial de quien la padece.

Algunas estrategias como el uso de faja o cirugía plástica dejan ver que la obesidad es reconocida inicialmente como un problema estético, donde el foco no se encuentra en resolver el problema de salud sino en mejorar la apariencia corporal para vivir una vida más plena; por lo que la cirugía bariátrica representa una oportunidad no solo de mejorar la salud, sino que es congruente con su anhelo de transformar su apariencia corporal de manera drástica en corto tiempo.

Contribuciones a disciplina: El reconocimiento de este fenómeno permite a los profesionales de enfermería comprender la obesidad desde la perspectiva de quien la padece, lo que contribuye en el desarrollo de intervenciones de cuidado centradas en la persona.

## Data Availability

Todo el conjunto de datos que apoya los resultados de este estudio fue publicado en el propio artículo.
